# Supplementary material for: Distinct impact of IgG subclass on autoantibody pathogenicity in different IgG4-mediated diseases
Source: eLife. 2022 Aug 3;11:e76223. doi: 10.7554/eLife.76223 (PMC9385207; doi:10.7554/eLife.76223)
Supplement: Supplementary file 1. — (a) Affinities of mouse FcγRs for human IgG4, IgG1, and its variants. (b) FcγR-binding properties of human IgG1 and its variants. (c) Demographic characteristics and laboratory findings of thrombotic thrombocytopenic purpura (TTP) patients. (d) Demographic characteristics and laboratory findings of pemphigus foliaceus (PF) patients. (e) Characteristics of anti-Dsg1 mAbs. (f) Polymerase chain reaction (PCR) primers used for cloning Dsg1, Dsg2, and Dsg1/Dsg2 chimeric molecules. [file elife-76223-supp1.docx]

**Supplementary Materials**

**Distinct impact of IgG subclass on autoantibody pathogenicity in different IgG4-mediated diseases**

Yanxia Bi^1,2^, Jian Su^3,4^, Shengru Zhou^5^, Yingjie Zhao^1,2^, Yan Zhang^1,2^, Huihui Zhang^1,2^, Mingdong Liu^1,2^, Aiwu Zhou^2^, Jianrong Xu^2^, Meng Pan^5*^, Yiming Zhao^3,4*^, Fubin Li^1,2,6*^

^1^Shanghai Institute of Immunology, Faculty of Basic Medicine, Shanghai Jiao Tong University School of Medicine, Shanghai 200025, China

^2^Key Laboratory of Cell Differentiation and Apoptosis of Chinese Ministry of Education, Shanghai Jiao Tong University School of Medicine, Shanghai 200025, China

^3^Jiangsu Institute of Hematology, The First Affiliated Hospital of Soochow University, Suzhou 215006, China

^4^Collaborative Innovation Center of Hematology, Soochow University, Suzhou 215006, China

^5^Department of Dermatology, Rui Jin Hospital, Shanghai Jiao Tong University School of Medicine, Shanghai 200025, China

^6^Leading contact: Fubin Li (fubin.li@sjtu.edu.cn)

^*^Address correspondence to Fubin Li (0086-18918925314; fubin.li@sjtu.edu.cn), Yiming Zhao (0086-13862045330; zhaoyimingbox@163.com), or Meng Pan (0086-13601698637; pm10633@rjh.com.cn)

**Supplementary Tables:**

**Supplementary file 1a. Affinities of mouse FcγRs for human IgG4, IgG1 and its variants.**

|  | FcγRI | FcγRIIB | FcγRIII | FcγRIV | Reference paper |
| --- | --- | --- | --- | --- | --- |
| IgG1 | 1(0.15μM) | 1(9.92μM) | 1(30.06μM) | 1(0.41μM) | a-d |
| IgG4 | 0.62-0.80 | < 0.90 | < 1.43 | < 0.02 | a,c |
| GASDALIE | 0.19-1.52 | 3.54-76.28 | 7.16-3005.75 | 2.07-413.00 | b,d |
| N297A | n. d. | n.d. | n.d. | n.d.b. | e |

n.d.b. indicates no detectable binding. n.d. indicates not done.

Reference paper:

a: Derebe, M. G., Nanjunda, R. K., Gilliland, G. L., Lacy, E. R., & Chiu, M. L. (2018). Human IgG subclass cross-species reactivity to mouse and cynomolgus monkey Fcgamma receptors. *Immunol Lett, 197*, 1-8. doi:10.1016/j.imlet.2018.02.006

b: Bournazos, S., Klein, F., Pietzsch, J., Seaman, M. S., Nussenzweig, M. C., & Ravetch, J. V. (2014). Broadly neutralizing anti-HIV-1 antibodies require Fc effector functions for in vivo activity. *Cell, 158*(6), 1243-1253. doi:10.1016/j.cell.2014.08.023

c: Dekkers, G., Bentlage, A. E. H., Stegmann, T. C., Howie, H. L., Lissenberg-Thunnissen, S., Zimring, J., . . . Vidarsson, G. (2017). Affinity of human IgG subclasses to mouse Fc gamma receptors. *MAbs, 9*(5), 767-773. doi:10.1080/19420862.2017.1323159

d: Gunn, B. M., Lu, R., Slein, M. D., Ilinykh, P. A., Huang, K., Atyeo, C., . . . Alter, G. (2021). A Fc engineering approach to define functional humoral correlates of immunity against Ebola virus. *Immunity, 54*(4), 815-828 e815. doi:10.1016/j.immuni.2021.03.009

e: Nimmerjahn, F., Bruhns, P., Horiuchi, K., & Ravetch, J. V. (2005). FcgammaRIV: a novel FcR with distinct IgG subclass specificity. *Immunity, 23*(1), 41-51. doi:10.1016/j.immuni.2005.05.010

**Supplementary file 1b. FcγR binding properties of human IgG1 and its variants ^(f, g)^**

| IgG1&variants | Fc mutation | Activating | | Inhibitory |
| --- | --- | --- | --- | --- |
|  |  | FcγRIIa | FcγRIIIa | FcγRIIb |
| IgG1 | - | + | + | + |
| GASDALIE | G236A/S239D/A330L/I332E | +++ | +++ | + |
| N297A | N297A | - | - | - |

f: Bournazos, S., DiLillo, D. J., Goff, A. J., Glass, P. J., & Ravetch, J. V. (2019). Differential requirements for FcgammaR engagement by protective antibodies against Ebola virus. *Proc Natl Acad Sci U S A, 116*(40), 20054-20062. doi:10.1073/pnas.1911842116

g: Sazinsky, S. L., Ott, R. G., Silver, N. W., Tidor, B., Ravetch, J. V., & Wittrup, K. D. (2008). Aglycosylated immunoglobulin G1 variants productively engage activating Fc receptors. *Proc Natl Acad Sci U S A, 105*(51), 20167-20172. doi:10.1073/pnas.0809257105

**Supplementary file 1c. Demographic characteristics and laboratory findings of TTP patients**

| Sex, F/M | 22/21/1^A^ |
| --- | --- |
| Median age, years (range) | 57 (5-83) |
| ADAMTS13 Ac (< 5%) /n | 44/44 |
| TTP | 1.3 (0.6-2.7)% |
| HC | 79.2 (52-105)% |
| ADAMTS13 Ag (ng/ml) |  |
| TTP | 28 (0-178) |
| HC | 487 (306-658) |
| IgG1 dominant TTP/n | 20/44 |
| ADAMTS13 Ac% | 1.274 (0.808-1.781) |
| ADAMTS13 Ag (ng/ml) | 12.535 (0-101.572) |
| IgG4 dominant TTP/n | 24/44 |
| ADAMTS13 Ac% | 1.384 (0.595-2.674) |
| ADAMTS13 Ag (ng/ml) | 40.953 (2.270-178.372) |

^A^Basic information of 1 TTP patients is incomplete.

**Supplementary file 1d. Demographic characteristics and laboratory findings of PF patients**

| Sex, F/M | 18/35 |
| --- | --- |
| Median age, years (range) | 62 (29-92) |
| αDsg1 unit value (U/ml) 1:100 | 191 (79-354) |
| Active | 197 (79-315) |
| Stable | 194 (101-354) |
| αDsg1 IgG unit value (U/ml) 1:1000 | 723 (260-2830) |
| Active | 1039 (284-2830) |
| Stable | 646 (260-1357) |
| αDsg1 IgG1 unit value (U/ml) 1:100 | 42 (2-188) |
| Active | 62 (6-188) |
| Stable | 35 (2-116) |
| αDsg1 IgG4 unit value (U/ml) 1:1000 | 538 (0-1338) |
| Active | 633 (0-1338) |
| Stable | 416 (8-1046) |

**Supplementary file 1e. Characteristics of anti-Dsg1 mAbs**

| Clone name | Heavy chain | | | VL | IIF/human | Pathogenicity | | Dsg1 epitope |
| --- | --- | --- | --- | --- | --- | --- | --- | --- |
|  | V | D | J |  |  | human | mouse |  |
| PF1-8-15 | VH3-30 | D5-24 | Jh4b | 3h | + | + | + | 89-101aa |
| PF24-9 | VH3-53 | D4 | Jh4b | 1c | + | + | + | 1-161aa |
| PF1-2-22 | VH1-08 | D3-3/DXP4 | Jh6b | O12/O2 | + | - | - | 1-161aa |

**Supplementary file 1f. PCR primers used for cloning Dsg1, Dsg2, and Dsg1/Dsg2 chimeric molecules**

| Upstream fragments | | | Downstream fragments | | | Dsg1/Dsg2 chimeric molecules | | |
| --- | --- | --- | --- | --- | --- | --- | --- | --- |
| PCR Products | Primers | | PCR Products | Primers | | PCR Products | Primers | |
|  | F | R |  | F | R |  | F | R |
| Dsg1 | 1 | 2 | Dsg2 | 3 | 4 |  |  |  |
|  |  |  |  |  |  |  |  |  |
| Dsg1 EC1 | 1 | 5 | Dsg2 EC2-5 | 6 | 4 | EC1 | 1 | 4 |
| Dsg1 EC1-2 | 1 | 7 | Dsg2 EC3-5 | 8 | 4 | EC-2 | 1 | 4 |
| Dsg1 EC1-3 | 1 | 9 | Dsg2 EC4-5 | 10 | 4 | EC1-3 | 1 | 4 |
| Dsg1 EC1-4 | 1 | 11 | Dsg2 EC5 | 12 | 4 | EC1-4 | 1 | 4 |
|  |  |  |  |  |  |  |  |  |
| Dsg1 EC2-5 | 14 | 2 | Dsg2 EC1 | 3 | 13 | EC2-5 | 3 | 2 |
| Dsg1 EC3-5 | 16 | 2 | Dsg2 EC1-2 | 3 | 15 | EC-3-5 | 3 | 2 |
| Dsg1 EC4-5 | 18 | 2 | Dsg2 EC1-3 | 3 | 17 | EC4-5 | 3 | 2 |
| Dsg1 EC5 | 20 | 2 | Dsg2 EC1-4 | 3 | 19 | EC5 | 3 | 2 |
| Primer 1 | 5'-TTTGGATCCATGGACTGGAGTTTCTTCAGAGTAGTT | | | | | | | |
| Primer 2 | 5'-TTTGTCGACATGTACATTGTCTGATAACAAATCTTTGG | | | | | | | |
| Primer 3 | 5'-TTTGGATCCATGGCGCGGAGCCCGGGA | | | | | | | |
| Primer 4 | 5'-TTTGTCGACGCCCACATAGGAGTCATGCTGTGCTTCC | | | | | | | |
| Primer 5 | 5'-AACAAAGACATCCTGTGTAAACACTGGAGGGTTGTC | | | | | | | |
| Primer 6 | 5'-GACAACCCTCCAGTGTTTACACAGGATGTCTTTGTT | | | | | | | |
| Primer 7 | 5'-TTCAAGCACTTTATTTTCCATGTAAGGGATATTATC | | | | | | | |
| Primer 8 | 5'-GATAATATCCCTTACATGGAAAATAAAGTGCTTGAA | | | | | | | |
| Primer 9 | 5'-TGAGATGACGCTGCTTTTAAACACTGGGCCTTCAAT | | | | | | | |
| Primer 10 | 5'-ATTGAAGGCCCAGTGTTTAAAAGCAGCGTCATCTCA | | | | | | | |
| Primer 11 | 5'-TGCATCGTGACAGATTGTAGTGTTCGGCTCTGTATT | | | | | | | |
| Primer 12 | 5'-AATACAGAGCCGAACACTACAATCTGTCACGATGCA | | | | | | | |
| Primer 13 | 5'-TGCAAATGTAGCCATTGAGAACACTGGTTCGTTGTC | | | | | | | |
| Primer 14 | 5'-GACAACGAACCAGTGTTCTCAATGGCTACATTTGCA | | | | | | | |
| Primer 15 | 5'-GGTATATGAAGACTGTTCTACTACAGGTATATTGTC | | | | | | | |
| Primer 16 | 5'-GACAATATACCTGTAGTAGAACAGTCTTCATATACC | | | | | | | |
| Primer 17 | 5'-TGTCTTTGAACCTGGACGAAAATGAATGCCTTCTTT | | | | | | | |
| Primer 18 | 5'-AAAGAAGGCATTCATTTTCGTCCAGGTTCAAAGACA | | | | | | | |
| Primer 19 | 5'-AGTATTGGTAGTAATTTTCTGCACAGGCTCTATCAG | | | | | | | |
| Primer 20 | 5'-CTGATAGAGCCTGTGCAGAAAATTACTACCAATACT | | | | | | | |
